# Differential Expression of CRH, UCN, CRHR1 and CRHR2 in Eutopic and Ectopic Endometrium of Women with Endometriosis

**DOI:** 10.1371/journal.pone.0062313

**Published:** 2013-04-24

**Authors:** Aikaterini Vergetaki, Udo Jeschke, Thomas Vrekoussis, Eirini Taliouri, Luca Sabatini, Evangelia A. Papakonstanti, Antonis Makrigiannakis

**Affiliations:** 1 Department of Obstetrics and Gynecology, Medical School, University of Crete, Heraklion, Greece; 2 Department of Obstetrics and Gynecology, Innenstadt Campus, Ludwig Maximilians University of Munich, Munich, Germany; 3 Centre for Reproductive Medicine, St Bartholomew's Hospital, London, United Kingdom; 4 Department of Biochemistry, Medical School, University of Crete, Heraklion, Greece; University of Edinburgh, United Kingdom

## Abstract

Endometriosis is considered as a benign aseptic inflammatory disease, characterised by the presence of ectopic endometrium-like tissue. Its symptoms (mostly pain and infertility) are reported as constant stressors. Corticotropin releasing hormone (CRH) and urocortin (UCN) are neuropeptides, strongly related to stress and inflammation. The effects of CRH and UCN are mediated through CRHR1 and CRHR2 receptors which are implicated in several reproductive functions acting as inflammatory components. However, the involvement of these molecules to endometriosis remains unknown. The aim of this study was to examine the expression of CRHR1 and CRHR2 in endometriotic sites and to compare the expression of CRHR1 and CRHR2 in eutopic endometrium of endometriotic women to that of healthy women. We further compared the expression of CRH, UCN, CRHR1 and CRHR2 in ectopic endometrium to that in eutopic endometrium of women with endometriosis. Endometrial biopsy specimens were taken from healthy women (10 patients) and endometrial and endometriotic biopsy specimens were taken from women with endometriosis (16 patients). Τhe expression of CRH, UCN, CRHR1, and CRHR2 was tested via RT-PCR, immunohistochemistry and Western blotting. This study shows for the first time that CRH and UCN receptor subtypes CRHR1β and CRHR2α are expressed in endometriotic sites and that they are more strongly expressed (p<0.01) in eutopic endometrium of women with endometriosis compared to healthy women endometrium at the mRNA and protein level. CRH, UCN, CRHR1 and CRHR2 mRNA were also more highly expressed in ectopic rather than eutopic endometrium (CRH, UCN, CRHR2α: p<0.01, CRHR1β: p<0.05) and protein (CRH and UCN: p<0.05, CRHR1 and CRHR2: p<0.01) in women with endometriosis. These data indicate that CRH and UCN might play an immunoregulatory role in endometriotic sites by affecting reproductive functions such as decidualization and implantation of women with endometriosis.

## Introduction

Endometriosis is one of the most important benign chronic diseases affecting the 6–10% of women of reproductive age, being mainly associated with pelvic pain, adhesion formation and infertility. Endometriosis is characterised by the ectopic presence of endometrial stroma and epithelium [1], [2]. Although its pathogenesis is still unclear, endometriosis has been proven to be both an estrogen-dependent and a chronic inflammatory disease [3], [4]. In that context, endocrine/paracrine influences and immunological aspects have been investigated. Thus, several growth factors, cytokines, immune cells and hormones in eutopic and ectopic endometrium, are considered to be involved in the pathophysiology of endometriosis-related infertility [5].

CRH (Corticotropin Releasing Hormone) is a 41-amino acid neuropeptide, synthesised in the hypothalamus, regulating the hypothalamus-pituitary-adrenal axis [6], [7]. CRH expression and biological functions are mediated by its membrane receptors, CRH-R1 (α, β, γ, c-h) and CRH-R2 (α, β, γ) [8], [9], [10]. CRH receptors are also activated by other endogenous agonists, such as urocortin (UCN), which is a 40-amino acid peptide belonging to the corticotropin-releasing hormone family and is structurally related to CRH [11], [12]. Apart from the central nervous system, CRH and its receptors are expressed in several sites of the female reproductive system, including the endometrial glands, decidualized stroma, trophoblast, syncytiotrophoblast and placental decidua [13], [14]. Moreover, CRH and UCN are secreted at inflammatory sites, acting as proinflammatory factors. Reproductive CRH has been shown to serve as an autocrine and paracrine modulator and to participate in decidualization, embryo implantation and maintenance of human pregnancy [15]–[24]. In addition, CRH and UCN mRNA have been found to be expressed by endometriotic cells, while endometriotic lesions show a strongly positive staining reaction for CRH and UCN [25]. The expression and function of CRH and UCN have also been found to be impaired in eutopic endometrium of women with endometriosis [26]. These data suggest a crucial role of CRH and UCN in pathogenesis of endometriosis. However, the relative expression of CRH, UCN and their receptors in eutopic and ectopic endometrium of endometriotic women and in eutopic endometrium of healthy women and women with endometriosis has never been investigated. Additionally, CRH receptors and in particular their subtypes have never been identified in endometriotic lesions. In the current study, we have investigated the relative expression levels of these molecules in eutopic and ectopic endometrium of endometriotic and healthy women providing the first evidence for a potential role of CRH receptors in infertility profile of endometriotic women.

The aim of this study was i) to examine the expression of CRH, UCN, CRHR1, CRHR2 and their subtypes CRHR1β and CRHR2α at endometriotic sites, ii) to compare the expression of those receptor in eutopic endometrium of endometriotic and healthy women and iii) to evaluate and compare the expression of CRH, UCN and their receptors in endometriotic sites with those in eutopic endometrium of endometriotic women.

## Materials and Methods

### Tissue Sample Collection

Endometrial biopsy specimens (at secretory phase, as it was confirmed by the progesterone levels of the women) were taken from healthy women (10 patients) undergoing hysteroscopy for diagnostic reasons as their most recent menstrual cycles were characterised by spontaneous spotting haemorrhage. The outcome of their hysteroscopy showed that they were all healthy apart from 3/10 having small polyps. Endometrial and Endometriotic tissue biopsies (stage III and IV) at secretory phase, as it was confirmed by the progesterone levels of the patients, were obtained from 16 patients diagnosed with endometriosis on different sites (peritoneal nodule, rectovaginal nodule, rectouterine nodule, right and left ovarian cyst endometriosis, left and right uterosacral legiment nodule), sharing all the same pathology, in the Department of Obstetrics and Gynaecology, St Bartholomew's Hospital of Queen Mary University, London, UK (Research Ethics Committee Reference Number: 05/Q0604/44). This research protocol was approved by the Ethics Committee of Queen Mary University, London, UK. All participants provided their written informed consent to participate. It is important to notice that the most critical reproductive hormone levels of both healthy and endometriotic patients did not affect the outcome of our research protocols as they ranged among: FSH levels (day 3 of the menstrual cycle): healthy patients 6–8 mlU/ml, endometriotic patients 7–9 mlU/ml, E_2_ levels (day 3 of the menstrual cycle): healthy patients 45±7 pg/ml, endometriotic patients 50±11 pg/ml and Progesterone levels (day 21 of the menstrual cycle): healthy patients 17±2 ng/ml, endometriotic patients 15±3 ng/ml.

### Tissue Homogenisation - RNA Extraction- cDNA Synthesis

RNA was isolated from frozen endometrium of healthy women (10 patients). Eutopic endometrium and endometriotic tissue samples from 16 patients with endometriosis were homogenised in Trizol (Invitrogen, Carlsbad, USA). RNA was measured in a spectrophotometer by measuring ultraviolet absorbance at 260 nm and used for the cDNA synthesis according to the cDNA synthesis Kit (Thermoscript, Invitrogen, Carlsbad, USA).

### RT-PCR Detection of CRH, UCN, CRHR1β and CRHR2α

To quantify mRNA expression of CRHR1β and CRHR2α in eutopic endometrium of healthy and endometriotic women and CRH and UCN in endometrium and endometriotic tissue from 16 patients, reverse transcription PCR (RT-PCR) of CRH, UCN, CRHR1β and CRHR2α. was performed. Ten microliters of the amplification products (CRH: 413 bp, UCN: 146 bp, CRHR1β:554 bps, CRHR2α:322 bps) was separated on a 2% agarose gel and visualized by ethidium bromide staining. Human CRH primer sequences were as follows: Forward: 5′- CAC CCT CAG CCC TTG GAT TTC -3′, Reverse: 5′- GCC CTG GCC ATT TCC AAG AC -3′. UCN primer sequences were as follows: Forward: 5′- CAG GCG AGC GGC CGC G-3′, Reverse: 5′- CTT GCC CAC CGA GTC GAA T-3′. CRHR1β primer sequences were as follows: Forward: 5′- ATG GAC GCG GCA CTG CTC CA-3′, Reverse: 5′ – CAC GGC CTC TCC ACG AGG G-3′. CRHR2α primer sequences were as follows: Forward: 5′- GGC CAG GCT GCA CCC ATT G-3′, Reverse: 5′- TCG CAG GCA CCG GAT GCT C-3′. All primers were provided by VBC Biotech, Vienna, Austria. Placenta and myometrium homogenated total RNA was used as positive controls and GAPDH (primers for GAPDH were: forward: 5′-GCCACATCGCTCAGACACCA-3′and reverse: 5′-GATGACCCTTTTGGCTCCCC-3′) as a house keeping gene. Band intensities of mRNA of interest were normalized with band intensities of GAPDH and expressed as arbitrary units (a.u.).

### Immunohistochemical Analysis

Formalin-fixed, paraffin-embedded tissue sections (4 µm thick) of eutopic and ectopic endometrium from 16 patients were deparaffinized in xylene and rehydrated through graded concentrations of ethanol. Antigen retrieval (350 W, 3 cycles, 5 min each in citrate buffer: 10% citric acid mix –9 ml citric acid and 41 ml sodium citrate in 450 ml ddH_2_0) was followed. After inhibition of endogenous peroxidases with 3% H_2_O_2_ (5 min), unspecific antibody binding was blocked with 10% power block (BioGenex Lig DAB substrate Pack, BioGenex Laboratories Inc, Fremont, CA, USA) for 10 min. Serial sections were then incubated with primary antibodies against human CRH (1∶200, H-019-06, Rabbit Anti-Corticotropin Releasing Factor, Phoenix Pharmaceuticals, Belmont, USA) or UCN (1∶200, H-019-14, Rabbit Anti-Urocortin Serum, Phoenix Pharmaceuticals, Belmont, USA), overnight at 4°C. Both blocking as well as detection and visualization of staining were performed by using the BioGenex Supersensitive link-label Detection System (BioGenex Laboratories Inc, Fremont, CA, USA) followed by the BioGenex Lig DAB substrate Pack (BioGenex Laboratories Inc, Fremont, CA, USA), according to the manufacturer’s protocols. Finally the slides were counterstained with Mayer’s heamatoxylin (Dako, Carpinteria, CA, USA) for 3 min, washed in tap water and covered using Glycergel (Dako, Carpinteria, CA, USA). Negative controls were performed by replacing the primary antibody with normal rabbit or goat IgG as isotype control and placental tissue was used as negative tissue control. The sections were examined by light microscopy. The intensity and distribution of the staining reaction were evaluated by two blinded, independent observers, including a gynaecological pathologist, using the semiquantitative immunoreactive score (IRS). The IRS was calculated by multiplication of optical staining intensity including glandular and stromal staining (graded as 0 = no reaction, 1 = weak, 2 = moderate and 3 = strong staining) and the percentage of positive-stained cells (0 = no positive, 1 = <25% of the cells, 2 = 25–50% of the cells, 3 = 51–75% of the cells and 4 = >75% of the cells) (Table 1). The IRS score derived from both the glandular and the stromal staining of the tissues.

**Table 1:** IRS Score results.

Intensity of staining	Percentage of positive cells	IRS points-classification		
0 = no reaction	0 = no positive	0–1 = negative		
1 = weak staining	1 = <25% of the cells	2–3 = mild		
2 = moderate staining	2 = 25–50% of the cells	4–8 = moderate		
3 = strong staining	3 = 51–75% of the cells	9–12 = strongly positive		
	4 = >75% of the cells			
**IRS points-classification**	**CRH expression** **endometrium**	**CRH expression** **endometriosis**	**UCN expression** **endometrium**	**UCN expression** **endometriosis**
0–1 = negative	–	–	–	–
2–3 = mild	–	–	–	–
4–8 = moderate	14/16 (87,5%)	9/16 (69,2%)	15/16 (93,75%)	11/16 (68,75%)
9–12 = strongly positive	2/16 (12,5%)	7/16 (43,75%)	1/16 (6,25%)	5/16 (31,25%)

We have found that 7/16(43,75%) ectopic endometrium samples showed strong CRH expression and 9/16(69,2%) ectopic endometrium samples showed moderate CRH expression compared to 2/16(12,5%) eutopic endometrium samples of strong CRH expression and 14/16(87,5%) eutopic endometrium samples of moderate CRH expression. Concerning the UCN expression in eutopic and ectopic endometium of the same patients, we have found that 5/16(31,25%) ectopic endometrium samples showed strong UCN expression and 11/16(68,75%) ectopic endometrium samples showed moderate UCN expression compared to 1/16(6,25%) eutopic endometrium samples of strong UCN expression and 15/16(93,75%) eutopic endometrium samples of moderate UCN expression.

### Western Blot Analysis

100 µg of proteins were extracted from healthy women’s eutopic endometrium (10 patients) and eutopic endometrium and endometriotic tissue from endometriotic patients (16 patients), placenta and myometrium followed by SDS-PAGE analysis in 10% acrylamide gel, and electrotransfer onto a nitrocellulose membrane. The membrane was blocked in 5% skim milk powder in 0.1% Tris-buffered saline/Tween for 20 min. The membrane was then incubated with CRHRI (CRF-RI (V-14) goat polyclonal antibody, Santa Cruz Biotechnology, Bergheimer, Germany) or CRH RII (CRF-RII (C-15) goat polyclonal antibody, Santa Cruz Biotechnology, Bergheimer, Germany) at a dilution of 1∶1000, followed by incubation with the peroxidase-conjugated donkey antigoat IgG secondary antibody. GAPDH was used as a house keeping gene. Protein extracts from placenta and myometrium used as positive controls. The specificity of CRHR1 and CRHR2 polyclonal antibodies was confirmed by absorption with blocking peptides sc-12381P and sc-20550P (Santa Cruz Biotechnology) respectively. The blocking peptides for CRHR1 and CRHR2 antibody were mixed with the CRHR1 and CRHR2 antibodies with a five-fold (by weight) excess of blocking peptides and incubated overnight at 4°C. We had the same samples loaded twice so that we could cut the nitrocelluse membrane in two lanes having identical samples and one lane was incubated with the polyclonal antibody and the other one with the blocked antibody followed by followed by incubation with the peroxidase-conjugated donkey antigoat IgG secondary antibody. The lane which had been incubated with the blocked peptide had its staining disappeared was specific to the antibody. Band intensities of protein of interest were normalized with band intensities of GAPDH and expressed as arbitrary units (a.u.).

### Evaluation and Statistical Analysis

Western blot and RT-PCR gel bands were analysed via image analysis software (Scion Corporation, Release Beta 4.0.2, Frederick, MD, USA). Statistical analysis was performed using the unpaired two-tailed Student's *t*-test. Any statistical difference at p<0.05 was considered significant.

## Results

### 1. CRH, UCN, CRHR1 and CRHR2 Expression in the Endometriotic Sites

**Figure 1:**
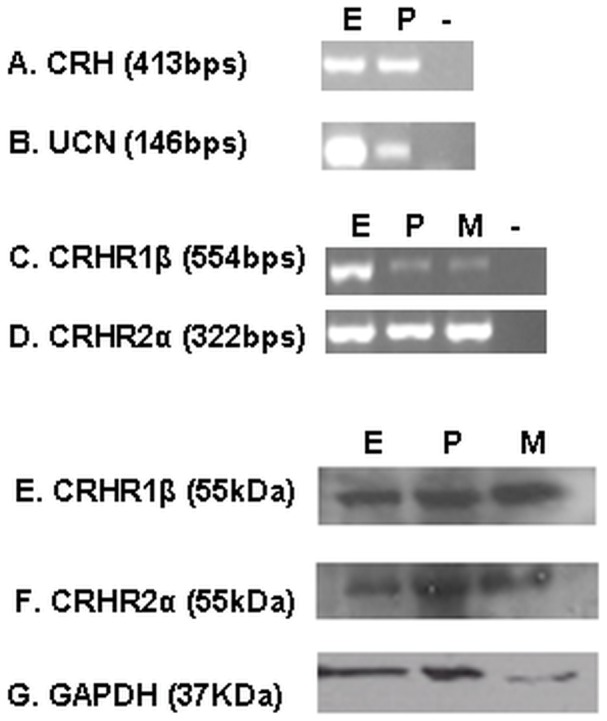
CRH, UCN, CRHR1 and CRHR2 expression in endometriotic sites. A: CRH (413 bps), B: UCN(146 bps), C: CRHR1β (554 bps) and D: CRHR2α (322 bps) mRNA expression and CRHR1 (55 kDa) and CRHR2 (55 kDa) protein expression in endometriotic tissue(E) (1E, 1F respectively), having placental tissue (P) and myometrial tissue (M) as positive control, negative sample(-), GAPDH(G) house keeping gene. (representative data).

In order to verify the expression of the CRH, UCN, CRHR1 and CRHR2 in endometriotic lesions, we firstly tested the expression of these transcripts by RT-PCR. Total RNA was extracted from endometriotic tissues obtained from sixteen patients with confirmed endometriosis. cDNA was synthesized and was screened for the presence of both the ligand transcripts (CRH and UCN) and their receptors’ transcripts (CRHR1β and CRHR2α). Both CRH and UCN genes were found to be transcribed in endometriotic tissues (Figure 1A, 1B). Interestingly, both CRHR1β and CRHR2α were also present in endometriotic tissues (Figure 1C, 1D). The above finding was further verified by evaluating the protein expression levels of CRHR1 and CRHR2 receptors in endometriotic sites. Total protein extracts from endometriotic tissues (obtained from 16 patients) were used for CRHR1 and CRHR2 detection by western blot. As shown in Figure 1E and 1F, both CRHR1 and CRHR2 are expressed in endometriotic sites. Placental and myometrial tissue were used as positive controls and GAPDH (Figure 1G) as a house keeping gene for Western blotting experiments.

### 2. High Expression Levels of CRHR1 and CRHR2 in Eutopic Endometrium of Endometriotic Women Compared to Eutopic Endometrium of Healthy Women

**Figure 2:**
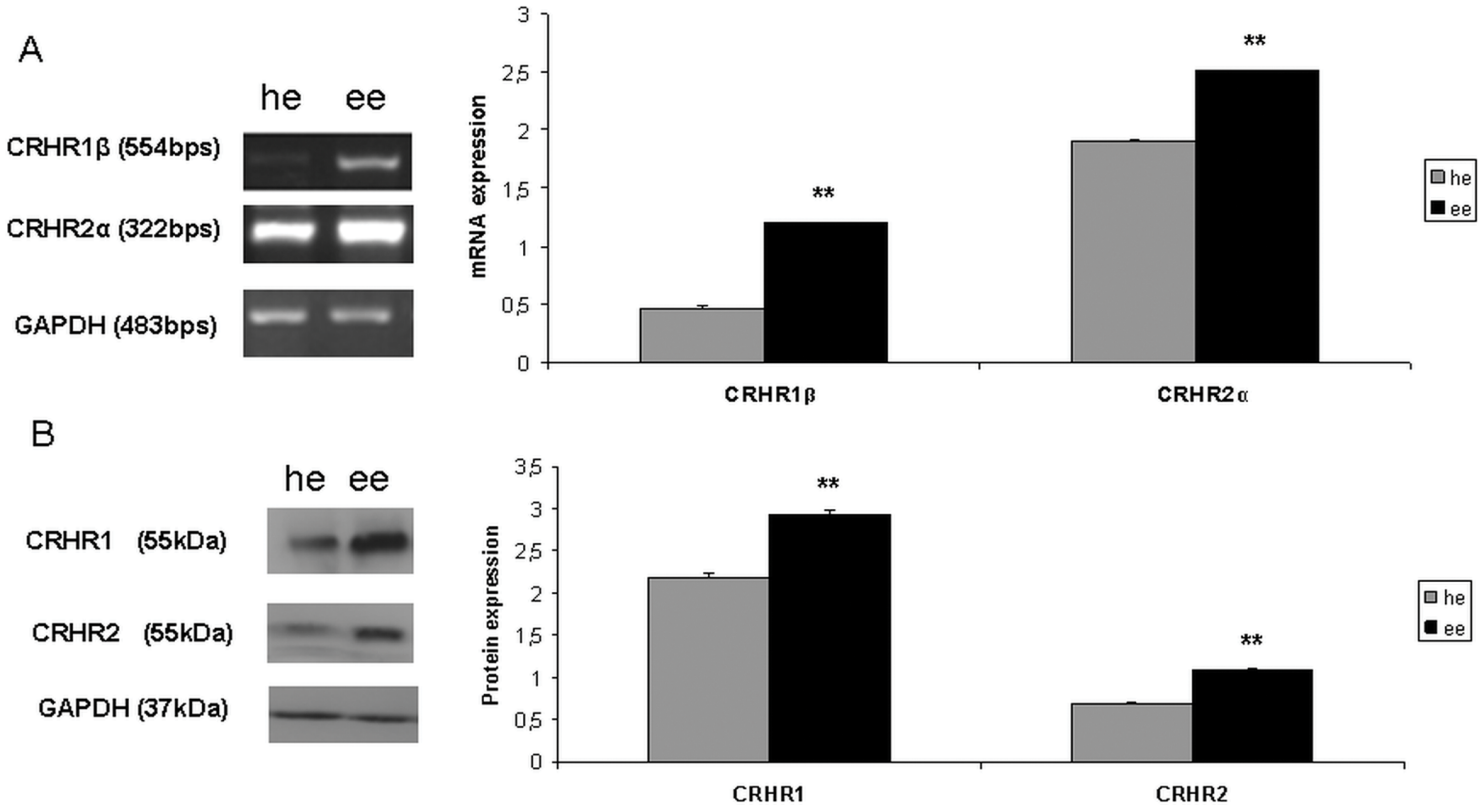
Higher CRHR1 and CRHR2 expression in eutopic endometrium of endometriotic women at an mRNA and protein level. A: mRNA expression of CRHR1β(554 bps) and CRHR2α(322 bps) is higher in eutopic endometrium of endometriotic women(ee) compared to eutopic endometrium of healthy women(he). B: protein expression of CRHR1 (55 kDa) and CRHR2 (55 kDa) is higher in ee compared to he. (**p<0.01) (representative data).

We then sought to evaluate and compare the expression of CRHR1 and CRHR2 in eutopic endometrium of both healthy and endometriotic women, at mRNA and protein level. mRNA extracts of eutopic endometrium of healthy women (10 patients) and endometriotic women (16 patients) used to perform RT-PCR for both receptors. We found that CRHR1β and CRHR2α are more highly expressed in eutopic endometrium of endometriotic women compared to eutopic endometrium of healthy women (CRHR1β:1.204±0.012 a.u. in endometriotic women vs 0.458±0.020 a.u. in healthy women, which corresponds to 2.62 fold increase when eutopic endometrium of healthy women is set as control, p<0.01; CRHR2α: 2.518±0.012 a.u in endometriotic women vs 1.895±0.016a.u. in healthy women, which corresponds to 1.32 fold increase when eutopic endometrium of healthy women is set as control, p<0.01) ) (Figure 2A). This was further corroborated at protein level (CRHR1: 2.926±0.048 a.u in endometriotic women vs 2.187±0.034 a.u in healthy women, which corresponds to 1.33 fold increase when eutopic endometrium of healthy women is set as control, p<0.01; CRHR2: 1.087±0.021 a.u in endometriotic women vs 0.685±0.017 a.u in healthy women, which corresponds to 1.58 fold increase when eutopic endometrium of healthy women is set as control, p<0.01) by performing western blotting for CRHR1 and CRHR2 (Figure 2B) using protein extracts from the same material. Both receptors showed an excessive expression in eutopic endometrium of endometriotic women compared to healthy women. GAPDH was used as a house keeping gene for both RT-PCR and Western Blotting.

### 3. CRH, UCN, CRHR1β and CRHR2α Molecules are more Abundant in Endometriotic Tissues Compared to the Corresponding Eutopic Endometrium

**Figure 3:**
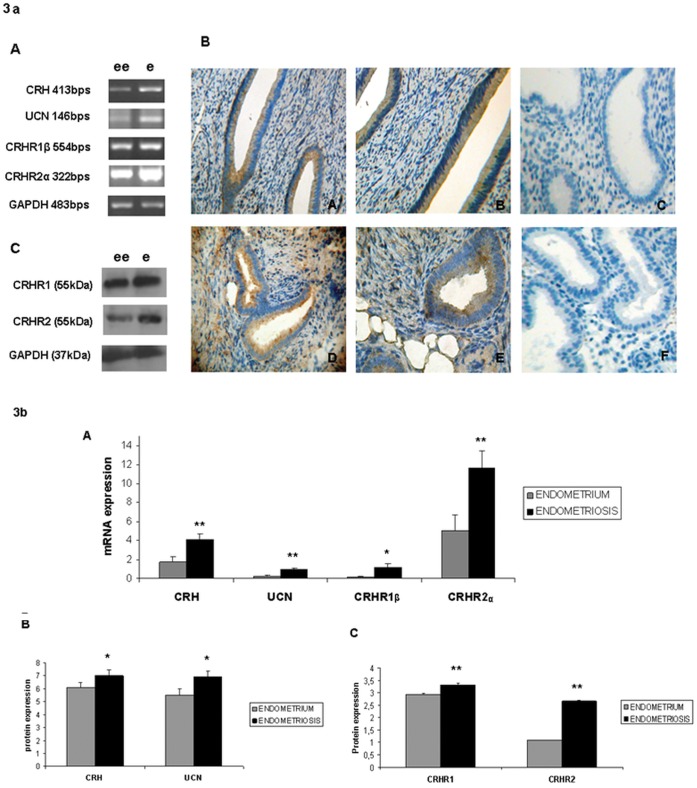
CRH, UCN, CRHR1 and CRHR2 excessive expression in endometriotic sites compared to eutopic endometrium of endometriotic women. **3a)** A: mRNA expression of CRH (413 bps), UCN (146 bps), CRHR1β (554 bps), CRHR2α (322 bps), GAPDH (483 bps) in endometriotic tissue (e) and the corresponding eutopic endometrium (ee). B: Immunohistochemical expression of CRH(A,D) and UCN(B,E) in eutopic(A,B) and ectopic endometrium(D,E). Both CRH and UCN are mainly expressed in endometriotic lesions.(C: negative eutopic endometrium, F: negative ectopic endometrium). C: Western blot immunodetection of CRHR1 (55 KDa) and CRHR2 (55 KDa), in endometriotic tissue(e) and eutopic endometrium(ee) of endometriotic women.GAPDH (37 kDa) used as a house keeping gene. CRH, UCN, CRHR1 and CRHR2α are significantly more expressed at an mRNA and protein level in endometriotic tissue compared to the corresponding eutopic endometrium. (representative data). 3b) Presentation of the normalized fold-increase of CRH, UCN, CRHR1 and CRHR2 expression in the study population at mRNA level(A) and protein level(B: CRH and UCN protein levels, C: CRHR1 and CRHR2 protein levels) (*, p<0.05, **,p<0.01).

We then compared the expression levels of CRH, UCN, CRHR1b and CRHR2a in the endometriotic lesions and the corresponding eutopic endometrium of the same women (16 patients). Total RNA was extracted from the eutopic and ectopic endometrial samples (obtained from 16 patients), cDNA was synthesized and was semi-quantitavely assessed for the presence of CRH, UCN, CRHR1β and CRHR2α transcripts. We found that in endometriotic foci CRH, UCN, CRHR1β and CRHR2α mRNA expression was significantly higher compared to the respective CRH, UCN, CRHR1β and CRHR2α mRNA content in the eutopic endometrium of the same patients (CRH: 4.101±0.582 a.u in ectopic vs 1.741±0.580 a.u in eutopic endometrium, 2.35 fold increase, p<0.01; UCN: 0.956±0.136 a.u in ectopic vs 0.282±0.075 a.u in eutopic endometrium, 3.38 fold increase, p<0.01; CRHR1β: 1.134±0.410 a.u in ectopic vs 0.186±0.046 a.u in eutopic endometrium, 6.07 fold increase, p<0.05; CRHR2α: 11.615±1.837 a.u in ectopic vs 5.023±1.723 a.u in eutopic endometrium, 2.31 fold increase, p<0.01. In all cases eutopic endometrium was set as control) (Figure 3a,A, 3b,A). The above findings were further verified by evaluating the protein expression levels in eutopic endometrium and endometriotic sites of the same women. The signal of CRH and UCN in western blot analysis was significantly low and could not be augmented because of the small size of the protein molecules, thus formalin-fixed paraffin-embedded tissue sections obtained from 16 patients were used for the detection of CRH and UCN by immunohistochemistry. According to IRS Score calculations (Table 1), CRH and UCN were found to be expressed more intensely in ectopic endometrium (Figure 3a,B(D) and Figure 3a,B(E) respectively) compared to eutopic endometrium (Figure 3a,B(A) and Figure 3a,B(B) respectively) of the same women (Figure 3b, B,) (CRH: 7.2±0.489 a.u in ectopic vs 6.1±0.378 a.u in eutopic endometrium, 1.18 fold increase, p<0.05; UCN: 6.9±0.458 a.u in ectopic vs 5.5±0.5 a.u in eutopic endometrium, 1.25 fold increase, p<0.05. In both cases eutopic endometrium was set as control). In order to identify the protein expression levels of the receptors CRHR1 and CRHR2 between eutopic and ectopic endometrium, western blot analysis was performed in 16 patients samples and showed that both receptors are more highly expressed in ectopic than eutopic endometrium of the same women (Figure 3a, C, Figure 3b, C) (CRHR1: 3.323±0.053 a.u in ectopic vs 2.926±0.048 a.u in eutopic endometrium, 1.13 fold increase, p<0.01; CRHR2: 2.657±0.040 a.u in ectopic vs 1.087±0.021a.u in eutopic endometrium, 2.44 fold increase, p<0.01. In both cases eutopic endometrium was set as control). GAPDH was used as a house keeping gene for both RT-PCR and western blotting analysis.

## Discussion

In this study, we show that CRH, UCN, CRHR1, CRHR2 and the receptor subtypes CRHR1β and CRHR2α are expressed in endometrium and endometriotic sites at mRNA and protein level. This is the first time that the receptor subtype expression is identified in endometriotic sites. Moreover, this study shows for the first time that CRHR1 and CRHR2 are more highly expressed in eutopic endometrium of endometriotic women compared to endometrium of healthy women. Interestingly, we found that the expression of CRH, UCN and their receptor subtypes, CRHR1 and CRHR2 is stronger in ectopic endometrium compared to that in eutopic endometrium of women with endometriosis.

Endometriosis is a disease characterized by increased stress. Both chronic pelvic pain and inflammation, commonly seen in endometriosis, act as stressors, supporting a deregulation of homeostasis towards a state of increased stress. Corticotropin releasing hormone (CRH) is the main hormone of stress, being expressed in different sites in the human body [11], [12]. Since endometriosis is a stress condition, it would be anticipated that reproductive CRH may play a key-role in pathophysiology of endometriosis, especially when pelvic pain or infertility are associated with the disease.

CRH and UCN are neuropeptides expressed in several sites of the female reproductive system including human endometrium [11], [12] and their highest mRNA expression is reached in the secretory phase [27], [13], [28]. The main source of endometrial CRH is epithelial cells whereas stromal cells express CRH only after the decidualization process has begun [13], [27], [28], [29]. CRH/UCN could activate mast cells triggering thus inflammation and adhesion formation, indicating an immune role of these molecules in eutopic endometrium. Moreover, these neuropeptides have been recently identified in endometriotic lesions [25], [26]. Concerning the CRH-related inflammatory profile of endometriosis, progestins could inhibit the CRH-induced inflammation of peritoneal cells *in vitro* [30]. Based on this published data, our study firstly examined and confirmed the CRH and UCN mRNA expression in endometriotic lesions of endometriotic women.

Given that CRH and UCN expression is mediated through CRHR1 and CRHR2 and these receptors are expressed in several sites of the female reproductive system [8], we investigated their expression in endometriotic lesions. Despite the fact that CRH/UCN have been implicated in endometriosis – a fact also verified by our results, no data has been reported so far concerning the expression of CRHR1 and CRHR2, the CRH and UCN receptors. This is the first time a study shows that CRHR1 and CRHR2 and specifically the CRHR1β and CRHR2α receptor subtypes are expressed at mRNA and protein level, not only in the endometrium but also at endometriotic sites indicating a potential crucial role of CRH and UCN in endometriosis.

We compared the expression levels of CRHR1 and CRHR2 in eutopic endometrium of endometriotic women with that in eutopic endometrium of healthy women to further examine the implication of these molecules in endometriosis and the infertility profile of endometriotic women. We show, for the first time, that CRHR1 and CRHR2 are significantly more expressed in the eutopic endometrium of endometriotic women compared to eutopic endometrium of healthy women. As previously described, CRH, UCN and CRHR1 are expressed by human epithelial and stromal endometrial cells. Endometrial stromal decidualization is a process taking place in the luteal phase of the menstrual cycle, where CRH expression is higher and leads to the induction of endometrial stroma decidualization, stimulated by progesterone in a cAMP – dependent manner [19], [27], [28], [29], [31], [32]. Upon decidualization, CRH inhibits the production of PGE2 and stimulates IL-1 and IL-6 production in human endometrial stromal cells [24]. Endometriosis is an aseptic inflammatory process accompanied by altered immune-related cell functions such as accumulation of macrophages and increased expression of growth factors, cytokines and specifically interleukins IL-1 and IL-6 [4], [5], [33], [34]. Additionally, the expression of CRH in endometriosis has been correlated with proinflammatory responses influencing thus innate and acquired immune responses [35], [36]. These data indicate that CRH and UCN may be of importance in maintaining chronic inflammation and thus local stress. Such stress could be further correlated to the local symptoms often seen in endometriosis as increased infertility rates, affected by modulation of the decidualization process and improper function of endometrium in women with endometriosis. A recent study [26] has proposed that there is an impaired CRH and UCN expression in eutopic endometrium of endometriotic women compared to healthy women endometrium. In our study we evaluated the CRHR1 and CRHR2 mRNA and protein expression levels in eutopic endometrium of endometriotic and healthy women and we show for the first time that the expression of both receptors is more elevated in eutopic endometrium of endometriotic compared to that of healthy women. We could hypothesize, as it has been shown by previous studies, that the impaired expression of CRH and UCN in eutopic endometrium of endometriotic women [26] and the reduced capacity of CRH and UCN to induce *in vitro* decidualization of endometriotic women stromal cells [26] may contribute to further expression of their receptor to keep a proper function of endometrium in endometriotic women. Unidentified endometrial defects in endometriosis could also affect the expression of CRH and UCN resulting in increased expression of CRHR1 and CRHR2 acting as a regulatory mechanism to compensate for the reduced efficiency of proper endometrial function of endometriotic women. Despite the fact that little is known so far for the expression patterns of the CRH receptors in intrauterine tissue, these findings could be in cohort with other studies showning that long term stimulation of pituitary [37] and myometrial cells [38].with CRH can down-regulate its own CRHR1 receptor. Further studies are needed to further elucidate this controversial expression pattern between CRH, UCN and their receptors expression in eutopic endometrium of endometriotic women.

CRH and UCN exert an important role not only in decidualization but also in blastocyst implantation [31]. Invasion of the blastocyst in the decidualized endometrium needs to take place for a successful embryo implantation [22]. CRH is produced by maternal decidual cells and embryonic trophoblast and is implicated in the maternal- blastoyst immune “cross-talk” by stimulating the expression of FasL in invasive extravillous trophoblast and maternal decidual cells and by increasing the apoptosis of T-lymphocytes through FasL induction. Thus the graft vs host reaction from the maternal immune system to the fetus is prevented and this is mediated through CRHR1 [32]. Studies in mice models have also shown that blocking of CRHR1 results in implantation dysfunctions [39] reinforcing thus the role of CRH in embryo implantation. Our findings showing that CRHR1 and CRHR2 are more abundantly expressed in eutopic endometrium of endometriotic women might explain the fact that endometriotic women can be fertile, characterised as hypofertile but not completely sterile, as there is excessive expression of CRHR1 and CRHR2 overcoming thus the low levels of CRH and UCN leading to implantation.

We also compared the expression of CRH, UCN, CRHR1 and CRHR2 in ectopic endometrium of endometriotic women compared to their eutopic endometrium. For the first time we found that all these molecules are more highly expressed in ectopic rather than eutopic endometrium of the same patients at mRNA and protein level. The fact that these molecules are more highly expressed in ectopic endometrium indicate that their function outside the uterus might be strengthened, which may potentially contribute to implantation and pregnancy maintenance problems of women with endometriosis.

Given also that high levels of stress are correlated with the progression of endometriosis [40] combined with the fact that CRH is activated by high levels of stress [6], [7], [30], our results may explain the neuroendocrine vicious circle of stress, mediated by CRH and UCN which is expected to maintain a chronic inflammatory profile as well as infertility.

In conclusion, the current study shows for the first time that not only CRH and UCN but also CRHR1 and CRHR2 are expressed in endometriotic lesions and that CRH, UCN, CRHR1 and CRHR2 are significantly more abundant in endometriotic lesions than the corresponding eutopic endometrium of endometriotic women. We also show that these receptors are more highly expressed in eutopic endometrium of endometriotic women compared to healthy individuals. Our findings point to a new inflammatory modulator pathway in which CRH, UCN, CRHR1 and CRHR2 are involved acting by an autocrine/paracrine pathway in eutopic and ectopic endometrium potentially affecting the pathogenesis of this benign disease and infertility profile of endometriotic women. These results suggest that a new therapeutic intervention could potentially based on blockage of CRH, UCN and their receptors leading to the improvement of the quality of endometriotic women’s life. Further mechanistic experiments and experiments on appropriate models needs to be done in order to clarify this role and highlight a potential use of anti-CRHR1 and anti-CRHR2 treatment in endometriosis.
